# Developing a Library Accessibility Plan: A Practical Guide for Librarians

**DOI:** 10.5195/jmla.2021.1318

**Published:** 2021-10-01

**Authors:** JJ Pionke

**Affiliations:** 1 pionke@illinois.edu, University of Illinois at Urbana-Champaign, Urbana, IL

Marrall, in the preface of her book *Developing a Library Accessibility Plan: A Practical Guide for Librarians*, states that her overall goal for her work is that, “library professionals will understand how to inventory, prioritize, design, implement, and assess a comprehensive improvement plan for electronic, physical, and instructional and/or programming related accessibility issues within their library” (p. xi). In this, she succeeds beautifully.

The book is divided into three parts for a total of fourteen chapters. Part I covers a broad and very general introduction into what accessibility across multiple dimensions (electronic, physical, and programming) looks like, as well as provides definitions, statistics, and a discussion of pertinent laws.

Part II is arguably the heart of the book. These chapters focus on not only how to create an accessibility plan but also, and perhaps more importantly, how to get buy-in from stakeholders to make the plan more actionable. Marrall begins part II with a discussion of how she defines an accessibility plan: “The document articulates where the library currently is and identifies where it wants to be within three to five years. To be truly useful, the plan must also articulate the *how*” (p. 67). She then discusses how to create the plan with an included table that breaks down parts of what a plan should be, what goes in each part of the plan, and responsibilities for each part. In this chapter, and subsequent chapters in part II, she includes tables that are either well-articulated breakdowns of processes or are worksheets that can be photocopied or recreated so that readers have a basis to complete suggested tasks. In chapter 8, she focuses on community partnerships, which is not something we often think of when working on improving accessibility. She discusses several library types in a table and outlines what community partnerships can look like, why they are important, and how to maintain them. For instance, in the table under Academic Libraries, she points to student disability organizations. Working with and empowering student groups will help the library determine what services and spaces need accessibility remediation.

In part III, Marrall provides several case studies. Chapters 9, 10, and 11 focus largely on completing tasks. For example, chapter 10 is titled “Conducting an Electronic Accessibility Audit to Improve Library Web Presence.” Chapters 12 and 14 discuss the creation of policies, and chapter 13 is a case study on education of a disability topic for library employees. Part III is where the rubber meets the proverbial road. The reader sees how the concepts discussed, especially in part II, are then implemented. For example, in chapter 12, Marrall walks the reader through “Creating an Accessibility Response Plan for Employees” (p. 123). She discusses explicitly a plan that helps library employees understand what to do when a patron complains about an accessibility issue to ensure there is a continuity and homogeneity of response. In Table 12.6, she provides a sample response plan that can be easily adopted and adapted by any library. The case studies are invaluable for understanding how the previous concepts are implemented.

Marrall's monograph is clear, concise, and most importantly, useful. She does not bog the reader down in sidebars or extraneous information, and she acknowledges when there are other routes of concepts that might be useful. For example, when she discusses project management in chapter 5 (p. 68), she states that there are many ways to manage projects and the library will need to pick the best method for them, but that discussing project management methodology is outside the scope of the chapter. In this way, Marrall provides guidance on how to do something but does not direct the reader to do something in a certain way. This kind of thinking and writing allows for maximum adaptability for the reader without the reader feeling tied to a specific way of doing things that would not be a good fit for their library or situation. I highly recommend *Developing a Library Accessibility Plan: A Practical Guide for Librarians* for any library that is serious about creating spaces and services that are accessible for all people, especially those with disabilities.

